# Navigating the International System for Reporting Serous Fluid Cytopathology in pericardial effusion: A meta‐analysis

**DOI:** 10.1002/cncy.70091

**Published:** 2026-03-19

**Authors:** Asma Arshia, David Kalfert, Ivana Kholová

**Affiliations:** ^1^ Department of Pathology University of Michigan Ann Arbor Michigan USA; ^2^ Department of Otorhinolaryngology and Head and Neck Surgery, First Faculty of Medicine Charles University University Hospital Motol Prague Czechia; ^3^ Pathology Fimlab Laboratories Tampere Finland; ^4^ Pathology, Faculty of Medicine and Health Technology Tampere University Tampere Finland; ^5^ Department of Clinical Medicine, Pathology and Forensic Medicine University of Eastern Finland Kuopio Finland

**Keywords:** cytopathology, diagnostic accuracy, International System for Reporting Serous Fluid Cytopathology, meta‐analysis, pericardial effusion, pericardial fluid, risk assessment

## Abstract

**Background:**

The International System for Reporting Serous Fluid Cytopathology (TIS) provides a standardized framework for classifying serous fluid cytology into five diagnostic categories: nondiagnostic, negative for malignancy, atypical, suspicious for malignancy, and malignant. Although TIS has been widely adopted for pleural and peritoneal fluids, its application in pericardial effusion cytology remains limited. The objective of this study was to evaluate the use of TIS in pericardial effusion samples and estimate the associated risk of malignancy for each category.

**Methods:**

A systematic review was conducted according to PRISMA (Preferred Reporting Items for Systematic Reviews and Meta‐Analyses) guidelines. Studies published between 2013 and 2023 were identified through a comprehensive PubMed search. Eligible studies applied TIS to pericardial effusion cytology. The analysis excluded case reports, case reviews, abstracts, comparative studies, and non‐English publications. Furthermore, the authors omitted studies that did not explicitly categorize pericardial fluid samples using the TIS categorization. Ten studies met inclusion criteria, comprising 2976 pericardial fluid samples.

**Results:**

The pooled distribution across TIS categories were: nondiagnostic (2.9%), negative for malignancy (60.2%), atypical (5.4%), suspicious for malignancy (2.4%), and malignant (23.4%). Risk of malignancy estimates based on histologic confirmation were: 10.8% for nondiagnostic, 8.7% for negative for malignancy, 34.0% for atypical, 64.1% for suspicious for malignancy, and 78.6% for malignant categories.

**Conclusions:**

TIS effectively stratifies pericardial effusion cytology samples by malignancy risk. The progressive increase in the risk of malignancy across categories supports its diagnostic utility. However, substantial heterogeneity, particularly in the negative for malignancy and malignant categories, highlights the need for standardized reporting and further prospective validation of TIS.

## Abstract 2

For this systematic review of 10 studies (2976 pericardial effusion samples), the authors evaluated the International System for Reporting Serous Fluid Cytopathology in pericardial cytology. Pooled prevalence and risk‐of‐malignancy estimates confirmed that the system is a useful stratification tool while revealing heterogeneity and the need for standardized reporting and prospective validation.

## INTRODUCTION

The terminology used in cytopathology has evolved significantly over the past several decades to improve diagnostic clarity and clinical communication with early frameworks, such as the Papanicolaou (Pap) system, laying the groundwork.^1^ Subsequent organ‐specific systems, such as the Paris System for urinary cytology, the Milan System for salivary gland cytopathology, and the World Health Organization lung system, established tiered, risk‐aligned reporting.^2^, ^3^, ^4^ Building on this foundation, the International System for Reporting Serous Fluid Cytopathology (TIS) provides a five‐tier structure linked to the risk of malignancy (ROM) to standardize interpretation and management across serous effusions.^5^, ^6^

Although TIS has been widely applied to pleural and peritoneal effusions, its use in pericardial fluid cytology is still relatively limited and not well studied. Pericardial fluid specimens often present unique diagnostic challenges because of their typically low cellularity, the frequent presence of reactive mesothelial cells, and the limited availability of ancillary testing.^7^ These factors can complicate the interpretation of atypical or suspicious findings and may contribute to heterogeneity in category assignment across institutions. Because pericardial effusions are much less common than other types of serous fluids, large‐scale studies have been limited, making it harder to establish reliable risk‐of‐malignancy (ROM) estimates and fully evaluate how well TIS performs in this setting. To address these gaps, this meta‐analysis pools published data to summarize how frequently each TIS diagnostic category is assigned in pericardial effusions and to estimate the corresponding ROM for each tier, with the specific objective of assessing consistency across studies and identifying areas for which prospective standardization could add value.^5^, ^6^, ^7^

## MATERIALS AND METHODS

This systematic review was conducted in accordance with the PRISMA (Preferred Reporting Items for Systematic Reviews and Meta‐Analyses)^8^ guidelines to evaluate the application of TIS in pericardial effusion cytology in the published literature.

### Search strategy and information sources

A comprehensive literature search was performed using PubMed in August 2024, covering studies published between January 1, 2013, and December 31, 2023. The search terms included combinations of keywords such as *pericardial effusion*, *cytopathology*, *serous fluid*, and *the International System for Reporting Serous Fluid Cytopathology* (TIS). An additional manual screening of reference lists from relevant articles was also performed.

### Eligibility and exclusion criteria

Studies were included in this review if they were published in English, applied the TIS categorization specifically to pericardial effusion cytology, and reported original data with sufficient detail to extract diagnostic categories and/or calculate the ROM. Studies were excluded if they were case reports, case series, review articles, or conference abstracts; if they were comparative studies not focused on TIS; or if they did not explicitly categorize pericardial fluid using the TIS system. These criteria carry inherent risks of bias. Restricting inclusion to English‐language publications can generate language or publication bias; omitting small series and abstracts tends to favor larger or more selective data sets; and requiring explicit TIS use may select centers with particular diagnostic thresholds. Because most studies were retrospective, selection and verification bias and incomplete follow‐up are also concerns. We documented study design, reference standards, and available follow‐up, and we incorporated these limitations into the interpretation of pooled estimates.

### Study selection

**FIGURE 1:**
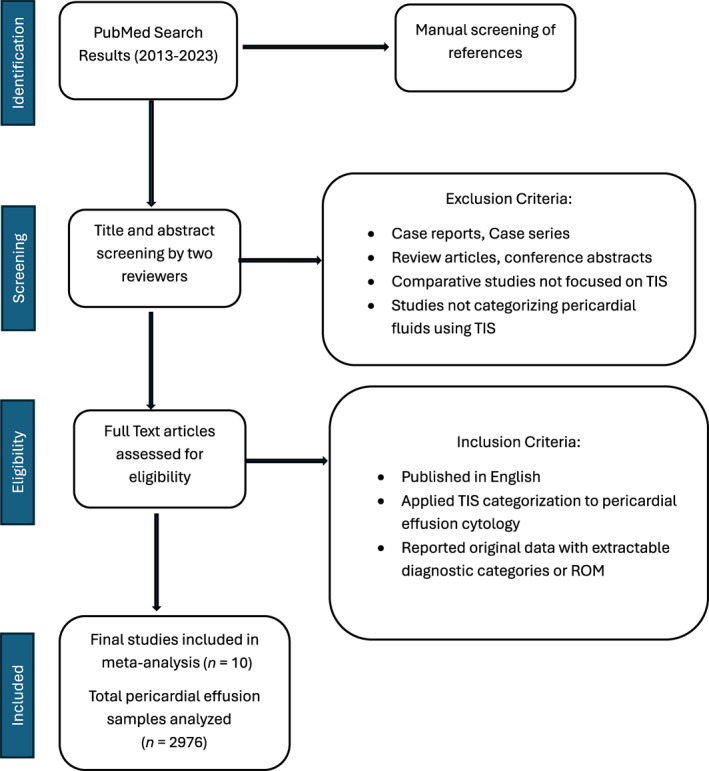
PRISMA flow diagram showing the selection of studies (*n* = 10) included in the meta‐analysis. Records were identified through PubMed and manual screening. PRISMA indicates Preferred Reporting Items for Systematic Reviews and Meta‐Analyses; ROM, risk of malignancy; TIS, the International System for Reporting Serous Fluid Cytopathology.

Two independent reviewers screened titles and abstracts for relevance. Full‐text articles of potentially eligible studies were retrieved and assessed against the inclusion criteria. Disagreements were resolved through discussion or consultation with the third reviewer. The selection process was documented using a PRISMA flow diagram (Figure 1).

### Data extraction

Data were extracted using a standardized form and included details such as study characteristics (including authors, year of publication, and country), sample size, and TIS categories applied. Information was also collected on the number of cases in each TIS category, the number of histologically confirmed cases and malignancies per category, the cytologic techniques used (such as staining and cell block preparation), and the use of ancillary methods, including immunocytochemistry and molecular testing.

### Statistical analysis

The pooled prevalence of each TIS category in pericardial effusion cytology was calculated based on data from multiple studies. The results are presented with 95% confidence intervals (CIs). The heterogeneity index (I^2^) was used to describe the variation between studies. The ROM was calculated for each TIS category using histologically confirmed cases from the included studies. All values, including prevalence and ROM, are reported as percentages with corresponding CIs and I^2^ values.

The statistical analysis was performed using Open Meta (Analyst); Center for Evidence‐Based Medicine, Brown University. A binary random‐effects model was applied to pool proportions across studies using the DerSimonian–Laird method to estimate between‐study variance (τ^2^). Effect sizes were reported as pooled estimates with corresponding 95% CIs. Statistical heterogeneity was assessed using the Cochran Q test and the I^2^ heterogeneity index together with associated *p* values. For studies that contained zero events in one of the comparison groups, a continuity correction of 0.5 was applied according to the standard software settings. The results were displayed using a forest plot generated directly in the software.

## RESULTS

### Study characteristics

This meta‐analysis included 10 studies that applied TIS to 2976 pericardial effusion cytology samples. Each study contributed data to one or more of the five TIS diagnostic categories: nondiagnostic, negative for malignancy, atypical, suspicious for malignancy, and malignant.

Our analysis included research conducted in different parts of the world. Three were conducted in the United States,^7^, ^9^, ^10^ three in India,^11^, ^12^, ^13^ and one each in China,^14^ Korea,^15^ Singapore,^16^ and Portugal,^17^ representing research across North America, Asia, and Europe. Of these, nine studies were retrospective in design,^7^, ^9^, ^10^, ^12^, ^13^, ^14^, ^15^, ^16^ and only one was prospective.^11^ In terms of staining methods, Giemsa and Pap stains were applied together in three studies,^11^, ^12^, ^16^ whereas five studies used Pap stain alone.^7^, ^9^, ^10^, ^15^, ^17^ Immunocytochemistry was performed in three studies,^13^, ^14^, ^17^ whereas immunohistochemistry (IHC) was used in nine.^7^, ^9^, ^10^, ^11^, ^12^, ^13^, ^15^, ^16^ The number of studies and cases varied by category, as detailed in Table 1.

**TABLE 1:** Pooled prevalence and risk of malignancy for the International System for Reporting Serous Fluid Cytopathology diagnostic categories in pericardial effusion cytology.^a^

Category	TIS categories			TIS risk of malignancy		
	No. of studies	No. of all cases	Pooled prevalence	No. of studies	No. of all cases	Risk of malignancy
Nondiagnostic	6	1998	2.9% (95% CI, 0.5%–5.2%; I^2^ = 87.81%)	2	36	10.8% (95% CI, −9.4%, 30.9%; I^2^ = 73.31%)
Negative for malignancy	7	2090	60.2% (95% CI, 45.8%–74.5%; I^2^ = 97.59%)	5	751	8.7% (95% CI, 2.2%–15.3%; I^2^ = 92.5%)
Atypical	9	2963	5.4% (95% CI, 3.2%–7.6%; I^2^ = 84.93%)	5	116	34.0% (95% CI, 12.2%–55.9%; I^2^ = 81.99%)
Suspicious for malignancy	8	1941	2.4% (95% CI, 1.4%–3.3%; I^2^ = 38.29%)	4	43	64.1% (95% CI, 41.9%–86.2%; I^2^ = 49.53%)
Malignant	10	2976	23.4% (95% CI, 17.1%–29.7%; I^2^ = 93.84%)	6	352	78.6% (95% CI, 59.8%–97.3%; I^2^ = 97.07%)

Abbreviations: CI, confidence interval; I^2^, heterogeneity index; TIS, the International System for Reporting Serous Fluid Cytopathology.

^a^ Summary of pooled data from 10 studies applying TIS to pericardial effusion samples. The table includes the number of studies and cases per category, pooled prevalence estimates, and corresponding risk of malignancy with 95% confidence intervals and heterogeneity indices.

### Distribution of TIS categories

**FIGURE 2:**
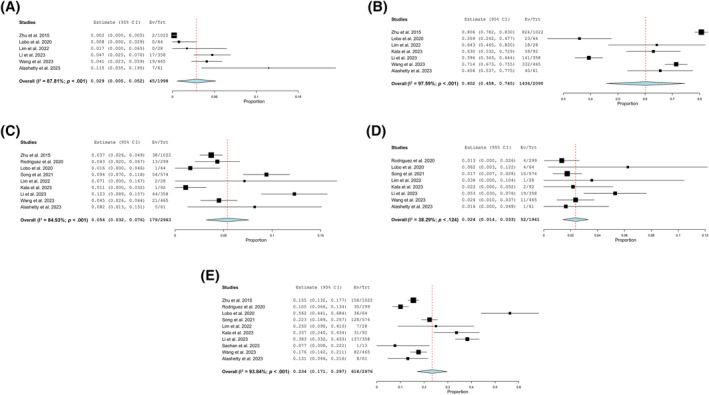
Pooled prevalence estimates for the International System for Reporting Serous Fluid Cytopathology diagnostic categories across included studies. The plots illustrate the pooled prevalence and heterogeneity for each category: (A) nondiagnostic, (B) negative for malignancy, (C) atypical, (D) suspicious for malignancy, and (E) malignant.CI indicates confidence interval; Ev/Trt, No. of events per treatment; I^2^, heterogeneity index.

The *nondiagnostic category* was reported in six studies, comprising a total of 1998 cases. The pooled prevalence was 2.9%, with a 95% CI of 0.5%–5.2% and substantial heterogeneity (I^2^ = 87.81%; Figure 2A). The *negative for malignancy category* was reported in seven studies, comprising 2090 cases. The pooled prevalence was 60.2% and was the most common category, with a 95% CI of 45.8%–74.5% and very high heterogeneity (I^2^ = 97.59%), the highest among all categories (Figure 2B). The *atypical category* was reported in nine studies, comprising 2963 cases. The pooled prevalence was 5.4%, with a 95% CI of 3.2%–7.6% and considerable heterogeneity (I^2^ = 84.93%; Figure 2C). The *suspicious for malignancy category* was reported in eight studies, comprising 1941 cases. The pooled prevalence was 2.4%, with a 95% CI of 1.4%–3.3% and moderate heterogeneity (I^2^ = 38.29%; Figure 2D). The *malignant category* was reported in all 10 studies, comprising 2976 cases. The pooled prevalence was 23.4%, with a 95% CI of 17.1%–29.7% and high heterogeneity (I^2^ = 93.84%; Figure 2E). Given the substantial between‐study heterogeneity (with I^2^ values very high for several categories), pooled prevalence and ROM estimates should be interpreted with caution; in this context, the principal value of the meta‐analysis is to demonstrate overall patterns and interstudy heterogeneity rather than to provide precise ROM benchmarks for clinical decision‐making.

### Risk of malignancy

**FIGURE 3:**
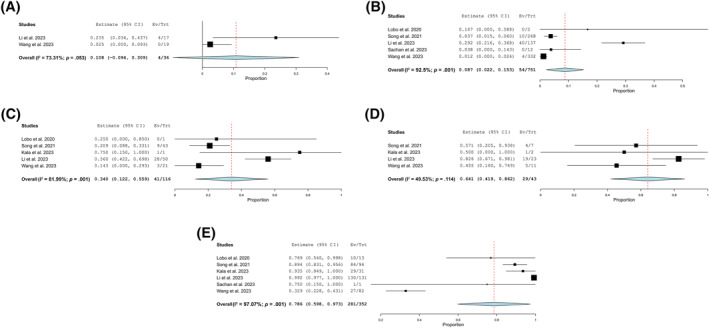
Pooled risk of malignancy estimates for the International System for Reporting Serous Fluid Cytopathology diagnostic categories across included studies. The plots illustrate the pooled risk of malignancy and heterogeneity for each category: (A) nondiagnostic, (B) negative for malignancy, (C) atypical, (D) suspicious for malignancy, and (E) malignant. CI indicates confidence interval; Ev/Trt, No. of events per treatment; I^2^, heterogeneity index.

The ROM values increased progressively across the TIS categories, from the lowest in the negative for malignancy group to the highest in the malignant category. The atypical and suspicious for malignancy categories showed intermediate ROM values. The ROM was calculated for each TIS category based on histologically confirmed malignancies. The numbers of studies and cases contributing to ROM estimates are illustrated in Figure 3.

The nondiagnostic category ROM was reported in three studies, comprising 36 cases. The ROM was 10.8%, with a 95% CI ranging from −9.4% to 30.9% and moderate heterogeneity (I^2^ = 56.19%; Figure 3A). The negative for malignancy category ROM was reported in five studies, comprising 751 cases. The ROM was 8.7%, with a 95% CI of 2.2%–15.3% and high heterogeneity (I^2^ = 92.5%; Figure 3B). The atypical category ROM was reported in five studies, comprising 116 cases. The ROM was 34.0%, with a 95% CI of 12.2%–55.9% and substantial heterogeneity (I^2^ = 81.99%; Figure 3C). The suspicious for malignancy category ROM was reported in four studies, comprising 43 cases. The ROM was 64.1%, with a 95% CI of 41.9%–86.2% and low heterogeneity (I^2^ = 49.53%; Figure 3D). The malignant category ROM was reported in six studies, comprising 352 cases. The ROM was 78.6%, with a 95% CI of 59.8%–97.3% and very high heterogeneity (I^2^ = 97.07%; Figure 3E).

## DISCUSSION

TIS was developed to bring consistency and clarity to the reporting of serous fluid cytology.^5^ Although its application has been increasingly studied in pleural and peritoneal effusions,^5^ its role in pericardial effusion cytology remains relatively sparsely studied. The objective of this meta‐analysis was to assess the utility of TIS in categorizing pericardial effusion samples and estimating the associated ROM across its five diagnostic categories.

Our analysis included 10 studies comprising 2976 pericardial effusion samples.^7^, ^9^, ^10^, ^11^, ^12^, ^13^, ^14^, ^15^, ^16^, ^17^ The studies we analyzed were conducted across several countries, highlighting the global interest in understanding and improving pericardial effusion cytology. Most cases were classified as negative for malignancy (60.2%), followed by malignant (23.4%), atypical (5.4%), suspicious for malignancy (2.4%), and nondiagnostic (2.9%).^7^, ^9^, ^10^, ^11^, ^12^, ^13^, ^14^, ^15^, ^16^, ^17^ The calculated ROMs indicated a progressive increase across the diagnostic spectrum, from 8.7% in the negative for malignancy category to 78.6% in the malignant category.^7^, ^9^, ^10^, ^11^, ^12^, ^13^, ^14^, ^15^, ^16^, ^17^ These findings are consistent with the intended stratification framework of TIS^6^, ^18^ and support its applicability in pericardial fluid cytology; and they indicate that the majority of pericardial effusion samples were classified as negative for malignancy, followed by the malignant and atypical categories. The high I^2^ values in several categories suggest substantial heterogeneity across studies.

The ROM for the atypical and suspicious for malignancy categories was 34.6% and 65.7%, respectively. When comparing the use of the atypical category across studies, institutional practice clearly varies. The pooled prevalence was 5.4% (95% CI, 3.2%–7.6%), and the high heterogeneity (I^2^ = 84.93%) suggests considerable variation. Song et al.^15^ and Li et al.^14^ reported higher proportions of atypical cases, which may reflect a cautious diagnostic approach or the presence of challenging specimens with borderline features. Li et al.^14^ also used an algorithmic method combining cytomorphology with immunocytochemistry, which likely increased the detection of subtle atypia. In contrast, Lobo et al.^17^ and Kala et al.^13^ reported lower use of the atypical category. Reported contributors include specimen quality, cellularity, and the adequacy of cell block material. Standardized TIS criteria and ancillary studies are described as tools to improve diagnostic consistency.^13^, ^17^ Beyond Li et al.,^14^ some studies support the integration of IHC in serous fluid cytology under the TIS framework. Sundling and Cibas^19^ emphasized the utility of markers like WT1, Claudin‐4, and BAP1 in distinguishing reactive mesothelial cells from malignancy, and Pinto et al.^6^ highlighted that incorporating molecular and IHC testing into TIS reporting can help refine intermediate category diagnoses and, in select patients, identify therapeutic targets.

Use of the suspicious for malignancy category also varies among institutions. The overall pooled prevalence from eight studies was 2.4% (95% CI, 1.4%–3.3%), with moderate heterogeneity (I^2^ = 38.29%), suggesting some heterogeneity in how this category was assigned. Lobo et al.^17^ reported a higher than average use of the suspicious category, which may be attributable to their oncology center setting. Studies by Rodriguez et al.,^9^ Song et al.,^15^ and Alashetty et al.^12^ demonstrated much lower use of this category, falling below the pooled average. Differences in diagnostic thresholds, laboratory practices, and specimen‐related factors, including quality, cellularity, and cell block adequacy, contribute to this heterogeneity.^9^, ^12^, ^15^ In our series, the ROM for suspicious for malignancy was 64.1%, whereas multiple TIS‐based studies spanning single‐center series and reviews often exceed 80%.^20^, ^21^ These findings highlight the need for clear diagnostic thresholds, proper training, and access to ancillary tests to support consistent and effective use across different centers.^20^, ^21^

The wide CIs and high heterogeneity observed in some categories, particularly negative for malignancy (I^2^ = 97.59%) and malignant (I^2^ = 93.84%), point to noticeable differences in how institutions interpret and report pericardial effusion cytology. Given the heterogeneity, ROM pooled estimates are best read as summaries of patterns and heterogeneity, not as precise benchmarks for clinical practice. We speculate that part of this heterogeneity reflects early phase differences in how standardized reporting systems are applied across institutions, similar to patterns described during the early use of The Bethesda System for Reporting Thyroid Cytology, in which differences in experience, training, and evolving criteria were associated with variable ROM estimates across centers.^22^

The negative for malignancy category, although the most frequently assigned diagnosis, demonstrated the highest heterogeneity. Interpretation of borderline findings varies across laboratories: some classify such cases as benign, whereas others designate them as atypical or suspicious based on similar cytologic features. These differences can be influenced by institutional protocols, access to ancillary testing, and the clinical context in which the cytology is interpreted.^7^, ^10^, ^12^, ^13^, ^14^, ^16^, ^17^ Radiologic correlation can be particularly valuable because it may prompt reassessment of the initial cytologic interpretation or further diagnostic workup when imaging findings conflict with cytologic interpretations.^19^

In contrast, the malignant category also showed high heterogeneity, despite its strong association with confirmed malignancy. One reason may be the inclusion of data from cancer centers and tertiary referral hospitals, in which the patient population tends to have a higher pretest probability of malignancy.^5^, ^11^, ^17^ These centers use advanced diagnostic tools, including immunocytochemistry and molecular testing, to support confident malignant classification. Consequently, their data may skew ROM estimates upward compared with general hospitals or community practices.^14^, ^16^, ^19^ Studies have demonstrated that tertiary institutions report higher ROM values and greater reliance on ancillary techniques, including cell block preparation and immunocytochemistry, to refine malignant diagnoses and reduce indeterminate categories.^5^, ^11^, ^14^, ^16^, ^17^, ^19^, ^23^

The limited number of prospective studies in our data set reflects the relatively recent implementation of the TIS system in pericardial effusion cytology. Among the included studies, only Sachan et al.^11^ specifically conducted a prospective analysis, focusing on effusion samples in an oncology setting. In contrast, the majority of studies, such as those by Rodriguez et al.,^9^ Song et al.,^15^ Li et al.,^14^ Wang et al.,^7^ Zhu et al.,^10^ Alashetty et al.,^12^ Lim et al.,^16^ Lobo et al.,^17^ and Kala et al.,^13^ were retrospective in nature, relying on reclassification of previously diagnosed cases. This imbalance is not unexpected given the novelty of TIS and the practical challenges of conducting prospective research in this area. Retrospective data are limited by selection bias and inconsistent follow‐up, reducing generalizability. Prospective, multicenter studies with standardized protocols are needed to more accurately evaluate TIS. Excluding non‐English publications further restricts representation of data from underrepresented regions. Limiting inclusion to English‐language reports introduces language and publication bias, and excluding small series or abstracts may overrepresent larger or more selective data sets. Restricting studies to those explicitly using TIS may also preferentially include centers with specific diagnostic thresholds. Because most available studies are retrospective, selection and verification bias and incomplete follow‐up remain concerns. We documented study design, reference standards, and available follow‐up and incorporated these limitations into interpreting pooled estimates.

Lagerstam et al. compared prospective and retrospective series applying the Milan System for Reporting Salivary Gland Cytology and concluded that the system demonstrates consistent diagnostic performance across both study designs, with prospective application offering slightly improved accuracy and ROM estimates.^24^

Despite these limitations, our findings support the diagnostic value of TIS in pericardial effusion cytology and, given interinstitutional heterogeneity, indicate a need to refine category definitions and strengthen training for consistent application.

Future studies should aim to validate these findings in larger, multicenter cohorts with standardized protocols. Prospective studies that correlate cytologic diagnoses with clinical outcomes and histologic follow‐up will be essential to refine ROM estimates and optimize the clinical utility of TIS. Within the TIS framework, incorporating ancillary techniques like immunocytochemistry and molecular testing could further improve diagnostic accuracy, particularly for the atypical and suspicious for malignancy categories. However, variations in staining practices, such as using Pap alone versus Pap with Giemsa and inconsistent application of immunocytochemistry and IHC across studies, may have influenced diagnostic categorization, underscoring the need for standardized approaches in evaluating pericardial fluid samples.

In conclusion, this meta‐analysis supports the conceptual validity of TIS in pericardial effusions, aligning with its established use in pleural and peritoneal fluids. Given the substantial heterogeneity in prevalence, ROM, and category thresholds across studies, pooled estimates should be interpreted cautiously, emphasizing trends rather than exact values. Future prospective, standardized investigations are warranted to clarify thresholds and improve the reliability of ROM estimates in this setting. The current ROM data demonstrate overall patterns and trends, not precise ROMs, for clinical management.

## AUTHOR CONTRIBUTIONS

**Asma Arshia**: Conceptualization, methodology, writing–original draft, data curation, formal analysis, writing–review and editing, and visualization. **David Kalfert**: Conceptualization, methodology, validation, software, data curation, formal analysis, visualization, writing–review and editing, and funding acquisition. **Ivana Kholová**: Conceptualization, resources, methodology, validation, writing–review and editing, and funding acquisition.

## CONFLICT OF INTEREST STATEMENT

The authors disclosed no conflicts of interest.
